# Risk and Criminogenic Needs of Youth Who Sexually Offended in Singapore: An Examination of Two Typologies

**DOI:** 10.1177/1079063213520044

**Published:** 2015-10

**Authors:** Gerald Zeng, Chi Meng Chu, Li Lian Koh, Jennifer Teoh

**Affiliations:** 1 Ministry of Social and Family Development, Singapore

**Keywords:** criminogenic needs, risk, youth sexual offending, typology

## Abstract

An increasing amount of research has been carried out to understand the characteristics of subgroups of adult sex offenders, but there is limited research into the risk factors and criminogenic needs of subgroups of youth who sexually offended. The current study investigated if there were differences in the risk and criminogenic needs of 167 Singaporean youth who sexually offended based on two typologies - youth who offended both sexually and nonsexually versus youth who offended only sexually, and youth who offended against child victims versus youth who offended against nonchild victims. Results show that youth who offended both sexually and nonsexually were found to have higher risk and criminogenic needs as compared to youth who only sexually offended. In addition, youth who offended against child victims were found to have higher numbers of previous sexual assaults as compared to youth who offended against nonchild victims. These differences have implications for the management and intervention of youth who sexually offended.

## Introduction

Sexual offending represents a serious issue for society that has severe and long-lasting consequences (Righthand & Welch, 2001); it has been estimated that sexual assault occurs to 1 in 6 adult women and 1 in 33 adult men (Center for Sex Offender Management, 2008). Although there has been substantial research into the risks factors and criminogenic needs of adult sexual offenders (Hanson & Morton-Bourgon, 2005), further research needs to be conducted on the risk factors for sexual recidivism and general criminogenic needs of youth who sexually offended.

### The Risk, Need, and Responsivity Framework

A way in which youth who sexually offended may be rehabilitated is through addressing their risk, need, and responsivity factors (RNR; Andrews, Bonta, & Hoge, 1990; Hanson, Bourgon, Helmus, & Hodgson, 2009). The RNR framework represents a set of principles that has been increasingly and successfully applied during offender intervention and rehabilitation (Andrews et al., 1990). Three principles are followed when applying RNR to rehabilitation: (a) the level and intensity of intervention should match the level of risk, that is, higher levels of service for high risk, lower levels of service for low risk; (b) intervention should target criminogenic needs that can be changed to reduce the risk of recidivism; and (c) the way in which intervention is delivered should be tailored to an offender’s individual learning style and abilities to ensure positive responsivity (Andrews & Bonta, 2010). The RNR framework has been applied successfully with adults who committed sexual offenses, and has been found to result in greater reductions in recidivism compared with interventions that did not incorporate RNR principles (Hanson et al., 2009). Similarly, lower levels of service matching among youth offenders have been found to be associated with earlier reoffending, as compared with higher levels of service matching (Vieira, Skilling, & Peterson-Badali, 2009), which was also found to predict a reduction in reoffending (Vitopoulos, Peterson-Badali, & Skilling, 2012). The RNR approach is thus a promising way to address youth sexual offending, the first step of which would be to identify the risk and criminogenic needs associated with youth sexual offending.

### Youth Who Sexually Offended and Criminal Diversity

As youth who sexually offended represent a heterogeneous population (Righthand & Welch, 2001; Sample & Bray, 2006), they may be classified into various subgroups to better understand the specific risk and criminogenic needs for each offending subgroup. This difference in risk and criminogenic needs would therefore translate to different assessment and treatment of each subgroup, which may consequently increase the responsivity to treatment and intervention for each subgroup of youth who sexually offended (Fanniff & Kolko, 2012).

One typology is that of youth who offended only sexually and youth who offended both sexually and nonsexually. The argument posited for classifying youth who sexually offended based on their criminal diversity is that youth who offend both sexually and nonsexually may have different risk and need factors that explain their sexual offending as compared with youth who offend only sexually (e.g., prior victim of sexual abuse, atypical sexual interests; Butler & Seto, 2002). Specifically, youth who commit only sexual offenses are more likely to have deviant sexual interests as compared with youth who commit both sexual and nonsexual offenses, but their levels of antisociality are comparable with that of nonoffenders (Butler & Seto, 2002). In contrast, youth who commit both sexual and nonsexual offenses are suggested to be more antisocial, as compared with youth who commit only sexual offenses (Butler & Seto, 2002).

Studies applying this typology in their investigations have established that there are differences in the risk and need profiles for both groups of youth who sexually offended. Butler and Seto (2002) found that youth who offended only sexually had fewer behavioral problems, more prosocial attitudes and beliefs, fewer childhood conduct problems, and lower expected risk of future delinquency as compared with youth who offended sexually and nonsexually. In addition, youth who offended sexually and nonsexually had sexually assaulted more unrelated victims as compared with youth who offended only sexually. Similarly, Aebi, Vogt, Plattner, Steinhausen, and Bessler (2012) found that youth who offended sexually and nonsexually were older at the time of first sexual offense, had more frequent general and violent recidivism, consumed drugs and alcohol in the context of such offending, and were more verbally aggressive.

Consistent with the abovementioned studies, Chu and Thomas (2010), after retrospectively coding for offender and offense characteristics, found that youth who offended sexually and nonsexually were less likely, as compared with youth who offended only sexually, to sexually offend against relatives. These youth were also found to be more likely to engage in violent recidivism, though the likelihood of sexual recidivism was similar for both groups. Likewise, Rajlic and Gretton (2010) found that youth who offended sexually and nonsexually had higher scores on two measures of risk of sexual recidivism (Estimate of Risk of Adolescent Sexual Offense Recidivism [ERASOR] and Juvenile Sex Offender Assessment Protocol–II [J-SOAP-II]), as compared with youth who offended only sexually. However, while total scores on both measures for youth who offended only sexually predicted sexual recidivism, total scores did not predict sexual recidivism for youth who offended both sexually and nonsexually (Rajlic & Gretton, 2010). These studies thus suggest that whether other nonsexual offending has been committed together with the sexual offending may be a meaningful typological distinction for youth who sexually offended.

### Youth Who Sexually Offended and the Age of Victims

The age of victims may also be used to classify youth who sexually offended. Such a classification stems from the postulation that adults who offended against child victims are distinct from adults who offended against adolescent or adult victims (i.e., nonchild victims). For example, studies have found that adults who sexually offended against nonchild victims had greater sexual aggression as compared with adults who sexually offended against child victims (Bard et al., 1987). In addition, adults who sexually offended against child victims have been found to possess more cognitive distortions about child sexual victimization than adults who offended against nonchild victims (Bumby, 1996), and to be less socially competent (Geer, Estupinan, & Manguno-Mire, 2000).

Some differences have been found between youth who sexually offended against child victims and those who offended against nonchild victims (see Fanniff & Kolko, 2012 for a review). For example, compared with youth who sexually offended against nonchild victims, youth who sexually offended against child victims have been found to score higher on the sexual drive and preoccupation scale of the J-SOAP-II (Parks & Bard, 2006), to be younger at the time of offense, abused more related and male victims, and to have engaged in more severe and intrusive offenses (Aebi et al., 2012). They have also been found to have higher rates of sexual recidivism (Kemper & Kistner, 2007). However, it has been suggested that many of these differences have not been found consistently across studies (Fanniff & Kolko, 2012). What has been consistently found though is that youth who sexually offended against nonchild victims are more likely to use force or violence and to offend against strangers (Fanniff & Kolko, 2012). Although this may be due to factors such as the increased tendency for a nonchild victim to resist, it may also possibly indicate that this subgroup of youth who sexually offended may possess greater general antisociality as compared with youth who sexually offended against child victims.

### Aims of the Present Study

The present study thus sought to examine if there were any differences in the risk and criminogenic needs of two typologies of youth who sexually offended: (a) youth who offended only sexually versus youth who offended sexually and nonsexually and (b) youth who sexually offended against child victims versus youth who sexually offended against nonchild victims. In particular, three hypotheses were examined: (a) that youth who offended sexually and nonsexually would be associated with higher levels of general risk and criminogenic needs as compared with youth who offended only sexually, (b) youth who sexually offended against child victims would have higher levels of risk of sexual recidivism as compared with youth who sexually offended against nonchild victims, and (c) youth who sexually offended against nonchild victims would have higher levels of general criminogenic risk and need as compared with youth who sexually offended against child victims.

## Method

### Sample

The sample consisted of 167^1^ male youth who sexually offended, aged 12 to 18 years (*M* = 14.90, *SD* = 1.43). The large majority of the sample were Chinese (44.3%, 74/167) or Malay (40.7%; 68/167); 12.0% (*n* = 20) were Indian, and 3% (*n* = 5) were of other ethnicity. A small minority of the sample (12.0%; 20/167) was also previously assessed by external psychological services, using the Wechsler Intelligence Scale for Children (Wechsler, 2004), to be functioning within the mental retardation range of intellectual ability. The sample comprised of all of the youth who were referred to the Clinical and Forensic Psychology Branch (CFPB) of the Ministry of Social and Family Development (Singapore) between October 2002 and December 2011 for a psychological assessment of their risk of future sexually abusive behavior. These youth were referred from a number of sources. All youth who sexually offended in Singapore are initially assessed by probation services, and child protection services (if applicable), for pre-sentencing as well as care and protection purposes, before referral to CFPB. In addition, youth who have committed sexual offenses while residing in youth correctional institutions are also referred to CFPB for sexual recidivism risk assessment. As such, the sample is representative of youth who have been charged or found to have committed sexual offenses in Singapore.

Almost three quarters (71.3%, 119/167) of the youth were referred from probation services, 20.4% (*n* = 34) were referred from youth correctional institutions, and 8.4% (*n* = 14) were from child protection services. Differences in age across these subsamples were nonsignificant. Of the total sample, more than three quarters (81.4%, 136/167) had committed molestation, 5.4% (*n* = 9) voyeuristic offenses, 10.8% (*n* = 18) exhibitionistic offenses, 14.4% (*n* = 24) nonconsensual fellatio, and 18.0% (*n* = 30) rape.

### Typologies of Youth Who Sexually Offended

Two typologies of youth who sexually offended were examined in this study; the first typology classified youth who sexually offended based on their criminal diversity. Youth who were charged and convicted with nonsexual offenses in addition to sexual offenses were classified as “youth who offended sexually and nonsexually.” Nonsexual offenses encompassed violent offenses (e.g., causing bodily harm, rioting, and robbery), and nonviolent and nonsexual offenses (e.g., theft, drug use, and fraud). Youth who were only charged and convicted with sexual offenses, and did not have any (prior or concurrent) nonsexual offenses were classified as “youth who offended only sexually.”

Among the 167 youth, 33.5% (56/167) committed nonsexual offenses in addition to sexual offenses and were classified as youth who offended sexually and nonsexually. Among these youth, 18 (32.1%) had committed violent offenses, and 38 (67.9%) had nonviolent nonsexual offenses. The remaining 111 (66.5%) youth who committed only sexual offenses were classified as youth who offended only sexually. The difference in age of referral between youth who offended only sexually (*M* = 14.95, *SD* = 1.46) and youth who offended sexually and nonsexually (*M* = 14.82, *SD* = 1.36) was nonsignificant.

A potential confound of the current research is that youth who have committed sexual and nonsexual offending have by default committed at least two offenses, whereas it is possible for youth who have committed only sexual offending to have committed only one offense. This may have a possible impact on the score for the Prior and Current Offenses domain of the Youth Level of Service/Case Management Inventory (YLS/CMI). The domain consists of five items that tap into prior convictions (three or more), prior failures to comply (two or more), prior probation, prior custody, and three or more current convictions. Thus, only the last item addresses the number of offenses that offenders commit. In the current study, the mean number of current offenses for youth who committed only sexual offending was 4.03 (*SD* = 5.37), whereas the mean number of current offenses for youth who committed both sexual and nonsexual offending was 6.21 (*SD* = 4.31). At the current means, both groups of youth would score the same on the last item (three or more current convictions) of the domain. In addition, both groups have approximately the same percentage of group members that have had two offenses—52.7% for youth who committed only sexual offending and 48.2% for youth who committed both sexual and nonsexual offending. It is thus unlikely that the categorization of youth who sexually offended based on criminal diversity will confound with the scores on the YLS/CMI domain for Prior and Current Offenses.

The second typology classified youth who sexually offended based on the age of their victims. Varying criteria used to classify offenders based on victim age (e.g., by the victim’s age alone, by the discrepancy between the offender and the victim’s age) have found little change in the differences identified between groups based on changes in classification criteria (Kemper & Kistner, 2010). The criterion used here was that defined in the ERASOR (described below), which defined a child victim as one being less than 12 years of age and at least 4 years younger than the youth who sexually offended (Worling & Curwen, 2001). It must be noted that all youth who offended against child victims were included in this subgroup. That is, youth who sexually offended against peer/adult victims *in addition* to sexually offending against child victims were categorized as “youth who sexually offended against child victims.”

Of the 167 youth, 26.9% (45/167) had victims who were children and were classified as youth who sexually offended against child victims. The remaining 122 (73.1%) youth had victims who were either peers or adults, and were thus classified as youth who sexually offended against nonchild victims. The difference in age of referral between youth who sexually offended against nonchild victims (*M* = 15.03, *SD* = 1.50), and youth who sexually offended against child victims (*M* = 14.56, *SD* = 1.16) was nonsignificant.

In terms of the typologies, 49.1% (82/167) of the sample consisted of youth who sexually offended against nonchild victims; 24.0% (40/167) had committed sexual offenses against nonchild victims but also had nonsexual offenses; 17.4% (29/167) had committed sexual offenses against child victims (17.4%, 29/167); and 9.6% (16/167) had committed sexual offenses against child victims but also had nonsexual offenses.

### Ethics

Ethical approval for the research was obtained through the Ministry of Social and Family Development.

### Youth Risk Assessment Measures

In addition to demographic information, the following measures were used to assess risk and criminogenic needs for the sample:

#### The Estimate of Risk of Adolescent Sexual Offense Recidivism (ERASOR)

The ERASOR (Worling & Curwen, 2001) is an empirically guided, structured clinical judgment measure that is designed to assist clinicians in estimating the risk of sexual recidivism for youth (aged 12-18 years) who have presented with sexual offending behaviors. It comprises 25 items (16 dynamic and 9 static risk factors), which can be coded as *unknown, not present, possibly/partially present*, or *present*, and which were assigned scores of 0, 1, 2, and 3, respectively, for purposes of statistical analysis. The items are grouped into five sections representing five risk domains for sexual recidivism: (a) Sexual Interests, Attitudes, and Behaviors, (b) Historical Sexual Assaults, (c) Psychosocial Functioning, (d) Family/Environmental Functioning, and (e) Treatment. However, the last domain of Treatment was excluded from all analyses because the youth who sexually offended in the sample had not received any treatment at the time of assessment. Based on the ratings for each item, evaluators make an overall clinical rating (i.e., structured professional rating/judgment) of *Low, Moderate*, or *High* risk. The current study examined domain and total scores that were derived from summing the scores for their respective items. The range of maximum and minimum scores for the overall ERASOR and its domains (without the Treatment domain—items 24 and 25) are displayed in Table 1. The ERASOR has been shown to have excellent reliability (e.g., intra-class correlation coefficients [ICCs] > .80 for total score and clinical judgment rating; Worling, Bookalam, & Litteljohn, 2012), and moderate predictive validity for predicting sexual recidivism (e.g., weighted area under curve [AUC] = .66 for both total score and clinical judgment rating; Viljoen, Mordell, & Beneteau, 2012). It has been validated in the Singaporean context (Chu, Ng, Fong, & Teoh, 2012).

**Table 1.** Range of Scores and Intra-Class Correlations for the ERASOR and YLS Domains.

Domains	Score range	ICC	ICC classification
ERASOR	0-69	.69	Good
Sexual interests, attitudes, and behaviors	0-12	.36	Poor
Historical sexual assaults	0-27	.73	Good
Psychosocial functioning	0-24	.58	Fair
Family/environmental functioning	0-12	.47	Fair
YLS	0-42	.76	Excellent
Prior and current offences/dispositions	0-5	.65	Good
Family circumstances/parenting	0-6	.58	Fair
Education/employment	0-8	.73	Excellent
Peer relations	0-4	.78	Excellent
Substance abuse	0-5	1.00	Perfect
Leisure/recreation	0-3	.55	Fair
Personality/behavior	0-8	.50	Fair
Attitudes/orientation	0-5	.46	Fair

*Note.* ERASOR = Estimate of Risk of Adolescent Sexual Offense Recidivism; YLS = Youth Level of Service; ICC = intra-class correlation coefficient.

#### The Youth Level of Service/Case Management Inventory 2.0 (YLS/CMI)

The YLS/CMI (Hoge & Andrews, 2010) is a structured assessment measure designed to facilitate the effective intervention and rehabilitation of youth (aged 12-18 years) who have committed criminal offenses by assessing their risk level and criminogenic needs. It comprises 42 items, with each item coded as either *absent* or *present*, and which were assigned scores of 0 and 1, respectively. All items are divided into eight domains: (a) Prior or Current Offenses/Dispositions, (b) Family Circumstances/Parenting, (c) Education/Employment, (d) Peer Relations, (e) Substance Abuse, (f) Leisure/Recreation, (g) Personality/Behavior, and (h) Attitudes/Orientation. The item scores can then be aggregated to obtain risk and criminogenic needs scores for each domain, as well as an overall score. Cutoff scores for *low, moderate*, and *high/very high* risk classifications are also available for each domain and the overall risk rating. Finally, the YLS/CMI contains a professional override feature that allows the above risk classification to be amended based on clinical judgment. However, as the YLS/CMI was used strictly as an actuarial measure in the present study, the risk classification and professional override features of the YLS/CMI were not examined. The range of maximum and minimum scores for the overall YLS/CMI and its domains are displayed in Table 1. The YLS/CMI has been found to have modest to good predictive validity for general, violent, and nonviolent recidivism among youth who have committed general offending (AUCs = .54-.74; Olver, Stockdale, & Wormith, 2009).The YLS/CMI was noted to have adequate predictive validity for violent and general (but not sexual) offending for assessing youth who sexually offended (AUCs = .61 and .66, respectively; Viljoen, Elkovitch, Scalora, & Ullman, 2009).

### Procedure

The current study was retrospective in nature, whereby five psychologists from the Ministry conducted clinical file reviews, and completed the YLS/CMI and ERASOR ratings for the current sample of youth who sexually offended based on file information. The clinical files contained (a) psychological reports prepared by psychologists at CFPB, (b) pre-sentencing reports prepared by probation officers, (c) institution risk and criminogenic needs reports, (d) charge sheets, (e) statement of facts, (f) any previously existing assessment and treatment reports on the youth’s CFPB files, as well as (g) school reports.

Demographic and offense-related information was coded from the files, including personal, family, psychiatric, and criminal offending histories as well as the current offending behaviors and risk management issues. Raters also coded the ERASOR and the YLS/CMI. To examine the inter-rater reliability for the measures, the five raters separately coded a randomly selected sample of 16 (9.6%) files. The ICCs and their corresponding classification for the ERASOR and YLS/CMI are displayed in Table 1. It must be noted that there was perfect agreement for the Substance Abuse domain score as all the youth who sexually offended in the inter-rater reliability sample was endorsed as not having any substance abuse issues (according to the coding criteria). As the ICC for the Sexual Interests, Attitudes, and Behaviors domain was poor (Cicchetti, 1994), this subscale was excluded from all subsequent analyses.

### Statistical Analyses

Descriptive statistics were first used to characterize the sample, with categorical data reported as numbers and percentages, and continuous data presented in terms of means and standard deviations. A two-way MANOVA was carried out to compare the characteristics of (a) youth who offended only sexually versus youth who offended sexually and nonsexually, and (b) youth who sexually offended against child victims versus youth who sexually offended against nonchild victims. The interaction between both the above typologies was also examined within the MANOVA. The independent variables entered into the MANOVAs were thus (a) the offense diversity and (b) the victim type, whereas the dependent variables were all four ERASOR and eight YLS/CMI domains. Analyses were conducted using SPSS version 19. A false discovery rate correction was used to control for the possibility of inflated Type I error as a result of the multiple post hoc comparisons conducted for the MANOVAs (Benjamini & Hochberg, 1995).

There is, however, one notable instance where items from the ERASOR may confound statistical differences. This is where Item 1 (“Deviant sexual interests in younger children, violence, or both”) of the Sexual Interests, Attitudes, and Behaviors domain, and Item 9 (“Ever sexually assaulted a child”) of the Historical Sexual Assaults domain would be endorsed for youth who sexually offended against child victims, but not for youth who sexually offended against nonchild victims. Therefore, within the victim-age-based typology, youth who sexually offended against child victims would have higher domain and total scores, due to automatic endorsements on these two items, as compared with youth who sexually offended against nonchild victims. To take this into account, the MANOVA was conducted with the abovementioned item scores being removed from their respective domain scores.

## Results

### Differences Between Risks and Criminogenic Needs Among the Typologies

The means and standard deviations of scores on the ERASOR and YLS/CMI for all the subgroups are displayed in Tables 2 and 3.

**Table 2.** Means and Standard Deviation for the Criminal Diversity Typology of Youth Who Sexually Offended on the ERASOR and YLS/CMI Domains.

	Sex-only (*n* = 111)		Sex-plus (*n* = 56)					
Domains	*M*	*SD*	*M*	*SD*	*df*	*F*	*p*	$\eta_{p}^{2}$
ERASOR								
Historical sexual assaults	12.65	3.01	12.64	2.96	1, 163	0.00	.982	.00
Psychosocial functioning	9.31	2.88	10.86	2.58	1, 163	5.54	.020^a^	.03
Family/environmental functioning	5.03	1.66	5.77	2.04	1, 163	1.87	.174	.01
YLS								
Prior and current offences/dispositions	0.51	0.76	1.12	0.92	1, 163	20.37	<.001^a^	.11
Family circumstances/parenting	1.90	1.45	2.68	1.50	1, 163	2.61	.108	.02
Education/employment	2.16	1.99	3.23	1.74	1, 163	6.08	.015	.04
Peer relations	2.35	1.48	3.11	1.22	1, 163	5.97	.016^a^	.04
Substance abuse	0.14	0.57	0.52	1.24	1, 163	4.16	.043	.03
Leisure/recreation	2.14	1.15	2.68	0.61	1, 163	7.39	.007^a^	.04
Personality/behavior	1.65	1.73	2.25	1.90	1, 163	2.10	.149	.01
Attitudes/orientation	0.87	1.16	1.20	1.03	1, 163	0.99	.322	.01

*Note.* ERASOR = Estimate of Risk of Adolescent Sexual Offense Recidivism; YLS/CMI = Youth Level of Service/Case Management Inventory.

a Significant after applying false discovery rate control.

**Table 3.** Means and Standard Deviations for the Victim-Age Typology of Youth Who Sexually Offended on the ERASOR and YLS/CMI Domains.

	Sex-only (*n* = 122)		Sex-plus (*n* = 45)					
Domains	*M*	*SD*	*M*	*SD*	*df*	*F*	*p*	$\eta_{p}^{2}$
ERASOR								
Historical sexual assaults	11.97	2.70	14.49	2.97	1, 163	25.10	<.001^a^	.13
Psychosocial functioning	9.94	2.98	9.51	2.56	1, 163	2.04	.155	.01
Family/environmental functioning	5.21	1.93	5.44	1.49	1, 163	0.00	.968	.00
YLS								
Prior and current offences/dispositions	0.67	0.86	0.84	0.88	1, 163	1.81	.181	.01
Family circumstances/parenting	2.09	1.52	2.36	1.46	1, 163	0.00	.981	.00
Education/employment	2.61	2.03	2.27	1.80	1, 163	2.31	.131	.01
Peer relations	2.61	1.51	2.60	1.23	1, 163	0.21	.647	.00
Substance abuse	0.32	0.97	0.13	0.46	1, 163	2.34	.128	.01
Leisure/recreation	2.34	1.04	2.24	1.00	1, 163	0.63	.427	.00
Personality/behavior	1.82	1.81	1.93	1.80	1, 163	0.00	.968	.00
Attitudes/orientation	0.98	1.10	0.98	1.22	1, 163	0.22	.641	.00

*Note.* ERASOR = Estimate of Risk of Adolescent Sexual Offense Recidivism; YLS/CMI = Youth Level of Service/Case Management Inventory.

a Significant after applying false discovery rate control.

#### Sexual offending versus sexual and nonsexual offending

There was a main effect of offense diversity on the MANOVA, *F*(11, 153) = 3.06, *p* = .001, $\eta_{p}^{2}$ = .18, indicating that youth who offended only sexually differed from youth who offended sexually and nonsexually on risk and needs domains. With regard to sexual recidivism risk factors (i.e., ERASOR ratings), tests of between-subjects effects revealed that youth who offended only sexually had significantly lower ratings of risk than youth who offended sexually and nonsexually in the domain of Psychosocial Functioning, *F*(1, 163) = 5.54, *p* = .020, $\eta_{p}^{2}$ = .03.

Pertaining to general criminogenic needs (i.e., YLS/CMI ratings), youth who offended only sexually had significantly lower ratings of risk than youth who offended sexually and nonsexually in the domains of Prior and Current Offenses, *F*(1, 163) = 20.37, *p* < .001, $\eta_{p}^{2}$ = .11; Peer Relations, *F*(1, 163) = 5.97, *p* = .016, $\eta_{p}^{2}$ = .04; and Leisure and Recreation, *F*(1, 163) = 7.39, *p* = .007, $\eta_{p}^{2}$ = .04.

#### Child victims versus nonchild victims

There was a main effect of victim type on the MANOVA, *F*(11, 153) = 2.92, *p* = .002, $\eta_{p}^{2}$ = .17, indicating that youth who sexually offended against child victims differed from youth who sexually offended against nonchild victims on risk and criminogenic needs domains. With regard to sexual recidivism risk factors, tests of between-subjects effects revealed that youth who sexually offended against child victims were rated significantly higher in risk than youth who sexually offended against nonchild victims in the domain of Historical Sexual Assaults (of the ERASOR), *F*(1, 163) = 25.10, *p* < .001, $\eta_{p}^{2}$ = .13. The differences between both groups in any of the criminogenic need domains of the YLS/CMI were nonsignificant.

#### Interaction

Multivariate tests of the interaction effect between criminal diversity and victim type was statistically nonsignificant, *F*(11, 153) = 1.16, *p* = .320, $\eta_{p}^{2}$ = .08.

## Discussion

The current study sought to ascertain if there were any differences in risk and criminogenic need profiles among two typologies of youth who sexually offended. Specifically, it compared the risk and need profiles of (a) youth who offended only sexually and youth who offended sexually and nonsexually, and (b) youth who sexually offended against child victims and youth who sexually offended against nonchild victims. Results indicated that there were significant differences in levels of sexual recidivism risk and general criminogenic needs among the groups in each typological classification.

### Youth Who Offended Only Sexually Versus Youth Who Offended Sexually and Nonsexually

Only about a third of the youth in the current study committed additional nonsexual offenses. This was consistent with some studies (e.g., 31.25% as cited in Butler & Seto, 2002), but was a much lower rate as compared with that found in previous studies (e.g., 89% to 94% as cited in Ronis & Borduin, 2007; 63% as cited in Ryan, Miyoshi, Metzner, Krugman, & Fryer, 1996). Such differences in prevalence rates may be cultural in nature, with the current results suggesting that the large majority of youth who sexually offended in Singapore tended to commit only sexual offenses during the initial offending, and as such, may differ in terms of sexual recidivism risk and general criminogenic needs from youth who do not sexually offend or youth who offended sexually *and* nonsexually.

Youth who offended sexually and nonsexually were found to present a higher risk of sexual recidivism in the domain of Psychosocial Functioning, as compared with youth who offended only sexually. Furthermore, youth who offended sexually and nonsexually were found to have higher levels of general criminogenic needs in the domains of Peer Relations and Leisure/Recreation as compared with youth who offended only sexually. There is thus support for the hypothesis that youth who offended sexually and nonsexually do differ in sexual recidivism risk factors and general criminogenic needs from youth who offended only sexually.

The finding that youth who offended sexually and nonsexually had higher general criminogenic needs as compared with youth who offended only sexually, suggests that general criminogenic risk and needs may influence the sexual offending committed by criminally diverse youth who sexually offended. These results may also be consistent with research that suggests that youth who offended sexually and nonsexually bear similarities to youth who offended nonsexually in their group characteristics (Butler & Seto, 2002). It may also be suggested that relationship and psychosocial functioning may represent particular salient risk factors and needs for youth who offended sexually and nonsexually, given that this subgroup was rated as having significantly higher risk for domains related to peer and psychosocial functioning, as compared with youth who offended only sexually. This is consistent with findings that items from the Psychosocial Functioning (antisocial interpersonal orientation, a lack of intimate peer relationships, interpersonal aggression) and Family/Environmental Functioning (problematic parent–child relationships) domains of the ERASOR have been found to be correlated with sexual recidivism (Worling et al., 2012).

The results in the current study are also consistent with research that has suggested that adult sex offenders differ in two general risk dimensions—sexual deviance (e.g., atypical sexual interests, excessive sexual preoccupation) and general antisocial orientation (e.g. antisocial personality, attitudes, and beliefs; Pullman & Seto, 2012). The risk for sexual or general (nonsexual) offending then varies based on the combination of these two domains—adult sex offenders high in sexual deviance are likely to sexually reoffend; those high in general antisocial orientation are likely to reoffend both sexually and generally, whereas those high in both sexual deviance and general antisocial orientation have the greatest likelihood of sexually reoffending (Pullman & Seto, 2012). Applying this to youth who sexually offended, it may be useful to assess their risk and needs along these two dimensions moving forward. However, further studies will need to ascertain whether the various combinations of both dimensions will bring about the suggested likelihoods of sexual and general reoffending.

### Youth Who Sexually Offended Against Child Victims Versus Youth Who Sexually Offended Against Nonchild Victims

Results indicated that the only difference in sexual reoffending risk factors between youth who offended against child victims, and youth who offended against nonchild victims was a greater number of previous sexual assaults for youth who offended against child victims. A possible explanation for this difference could be a greater availability of child victims, and a lower risk of a child victim being able to resist an older and stronger perpetrator. Furthermore, youth who offended against child victims did not differ significantly from youth who offended against nonchild victims in terms of level of general criminogenic needs. This is consistent with previous research that has also found general criminogenic factors such as aggression and impulsivity not to discriminate between offenders with child versus nonchild victims (Bard et al., 1987; Overholser & Beck, 1986).

However, the current finding that only the number of prior sexual offenses differentiates youth who offended against child victims from youth who offended against nonchild victims brings into question the meaningfulness of using a victim-age-based categorization for youth who sexually offended. A classification is only useful if there are significant differences between groups other than that which is expected of the subgroup itself. It is possible that the current study has not examined variables that have been found to be psychologically meaningful to the victim-age-based (i.e., child/nonchild) typology. Although prior research has produced mixed results as to whether both youth who sexually offended groups differ on variables such as sexual victimization (Fanniff & Kolko, 2012; Seto & Lalumière, 2010), mental health issues (Fanniff & Kolko, 2012; Ronis & Borduin, 2007), and low self esteem (Ford & Linney, 1995), the current study did not use such variables in its investigation. Such variables may prove to be meaningful for youth who sexually offended in a non-Western context, and should be explored in future studies. Another point to note is that it has been suggested that the victim-age-based typology for youth who sexually offended is based on research that has been conducted on adult sexual offenders (Fanniff & Kolko, 2012). Although studies have found differences between these adult subtypes, results here suggest that applying such a categorization to youth who sexually offended may not have as much utility.

### Limitations and Future Studies

In terms of limitations, it must be noted that the sample size for the current study was modest, which might have affected the power for some analyses. The modest sample size also limited the number of groups that could be generated and analyzed. Another limitation of the current study is the poor ICCs obtained for the Sexual Interests, Attitudes, and Behaviors domain of the ERASOR (ICC = .36); ICCs for the other four domains ranged from fair to good, and the ICC was good for the total score, and comparable with that found for other investigations (Viljoen et al., 2009; Worling, 2004; Worling et al., 2012). Poor ICC ratings for the domain may indicate that ratings among the five raters for the domain may not have been adequately reliable. Investigation of this particular domain may be important because it has been suggested that atypical sexual interests start to form early in life and strengthen if these interests are acted on (Hunter & Becker, 1994). Furthermore, it has been suggested that deviant sexual interests is one of several risk factors predicting sexual recidivism (Worling & Långström, 2006). Although previous studies have found adults and adolescents who offended against child victims have greater atypical sexual interests, greater sexual preoccupation, and cognitive distortions, compared with offenders with nonchild victims (Bard et al., 1987; Parks & Bard, 2006), this could not be ascertained in the current study because of the poor inter-rater reliability for the corresponding domain of the ERASOR. Further studies should therefore be carried out to ascertain if there are indeed differences in deviant sexual interests, attitudes, and behaviors between youth who sexually offended against child and nonchild victims.

Future studies with a larger sample size could also examine different groups of offenders within each subgroup. For example, criminally diverse youth who sexually offended could be further classified into youth who have committed additional violent or nonviolent offending in addition to their sexual offending. Similarly, youth who sexually offended against both child and nonchild victims could be grouped separately as a mixed group of youth who sexually offended against victims with a wide age range (e.g., Kemper & Kistner, 2007). Moreover, future studies could also seek to explore other categorizations of youth who sexually offended. For example, youth who sexually offended could be classified based on their developmental trajectory and their long-term outcomes could be examined (e.g., Pullman, Leroux, & Seto, 2012).

Another consideration is that although youth who offended both sexually and nonsexually have been found to possess greater antisocial risk and needs as compared with youth who offended only sexually, it is not clear whether such risk and needs are greater or lesser than that in youth who offended nonsexually. It could thus be worthwhile for future studies to apply the classifications here in a risk and needs comparison with youth who offended nonsexually, to identify similarities and differences in risk and needs for all these groups.

In summary, the current study has found very few differences in sexual and general risk and needs between youth who sexually offended against child victims and youth who sexually offended against nonchild victims. However, important differences in risks and needs pertaining to psychosocial functioning were found among criminally diverse youth who sexually offended as compared with youth who offended only sexually. Such findings should provide a greater understanding of youth sexual offending and may help support individualized assessment and treatment of youth who sexually offended.

## Notes

1. One individual was removed from the sample due to errors in the coding of the case file.
